# An Immune-Related Prognostic Signature Predicts Overall Survival in Stomach Adenocarcinomas

**DOI:** 10.3389/fgene.2022.903393

**Published:** 2022-05-23

**Authors:** Kangjie Zhou, Nan Hu, Yidong Hong, Xueyu Wu, Jingzhou Zhang, Huan Lai, Yang Zhang, Fenglei Wu

**Affiliations:** ^1^ Department of Oncology, Lianyungang Clinical College of Nanjing Medical University/The First People’s Hospital of Lianyungang, Lianyungang, China; ^2^ Hospital of Xuzhou Medical University, The First People’s Hospital of Lianyungang, Lianyungang, China; ^3^ Department of Oncology, The Affiliated Lianyungang Hospital of Xuzhou Medical University/The First People’s Hospital of Lianyungang, Lianyungang, China; ^4^ Department of Obstetrics and Gynecology, The Affiliated Lianyungang Hospital of Xuzhou Medical University/The First People’s Hospital of Lianyungang, Lianyungang, China

**Keywords:** Stomach adenocarcinomas, immune phenotype, immune infiltration, prognostic model, immunotherapy

## Abstract

This study aimed to explore an immune response-related gene signature to predict the clinical prognosis and tumor immunity of stomach adenocarcinomas (STAD). Based on the expression and clinical data of STAD in the TCGA database, the immune cell infiltration status was evaluated using CIBERSORT and ESTIMATE methods. Samples were grouped into “hot” and “cold” tumors based on immune cell infiltration status and consensus clustering. The infiltration abundance of activated memory CD4 T cells and CD8 T cells had a significant effect on the overall survival of STAD patients. Among the three clusters, cluster 2 had a higher immune score and a significantly higher abundance of CD8 T cells and activated memory CD4 T cells were assigned as a hot tumor, while cluster 1 and 3 were assigned as a cold tumor. DEGs between hot and cold tumors were mainly enriched in immune-related biological processes and pathways. Total of 13 DEGs were related to the overall survival (OS). After the univariate and multivariable Cox regression analysis, three signature genes (PEG10, DKK1, and RGS1) was identified to establish a prognostic model. Patients with the high-risk score were associated with worse survival, and the risk score had an independent prognostic value. Based on TIMER online tool, the infiltration levels of six immune cell types showed significant differences among different copy number statuses of PEG10, DKK1, and RGS1. In this study, an immune-related prognostic model containing three genes was established to predict survival for STAD patients.

## 1 Introduction

Stomach cancer is the fifth most common cancer worldwide and the third leading cause of cancer death (Bray et al., 2018). The new cases of gastric cancer in China account for 47% of the total number of gastric cancer in the world every year, and more than 60% of the patients are locally advanced or advanced at the time of treatment, and the 5-year survival rate is less than 30% (Chen et al., 2016; Feng et al., 2019). The annual death rate of gastric cancer in China has dropped from 3.8% to 2.3% over the past decade due to advances in diagnosis and treatment technology (Gao and Wu, 2019). Stomach adenocarcinomas (STAD) are the most common type of stomach carcinomas (Rima et al., 2020). Conventional treatments such as surgery, chemotherapy, and radiotherapy have limited efficacy for stomach cancer. Although molecularly targeted drugs such as Trastuzumab, Apatinib, and ramucirumab have been approved for stomach cancer successively, the targeted therapy of stomach cancer is still far behind lung cancer, breast cancer, colon cancer, and other common tumors (de Haas et al., 2014; Tabernero et al., 2018).

Tumor cells can escape the surveillance of immune response through various mechanisms, one of the most important mechanisms is immune checkpoint mediated co-inhibitory signaling pathway (Waldman et al., 2020). The high expression of various checkpoint proteins on T cells in stomach cancer tissues, including CTLA-4, IDO, LAG3, and PD-1, suggests that they have the phenotype of T cell immune exhaustion and the tumor microenvironment is in an immunosuppressive state (Taieb et al., 2018; Vrána et al., 2018; Li K. et al., 2021). Some stomach cancer tumor cells also have a high mutation load, especially those with high microsatellite instability, which can express abundant tumor antigens and thus initiate a stronger immune response (van Velzen et al., 2020). Moreover, checkpoint proteins PD-1 and PD-L1 were up-regulated in tumors with high microsatellite instability (Llosa et al., 2015), which showed well response to PD-1 inhibitors (Le et al., 2015). But not all stomach cancer patients benefit from immunotherapy alone. Therefore, it is necessary to explore the immunophenotypic classification of stomach cancer to screen the dominant population that may benefit from immunotherapy and to identify suitable biomarkers for monitoring treatment efficacy.

It has been demonstrated that the “hot” tumor phenotype has high response rates to immune checkpoint inhibitors, in which the immune cell infiltration in the tumor microenvironment are mainly characterized, while tumor with low immune infiltrations is regarded as “cold” tumor (Maleki Vareki, 2018). Such tumor classification can partly explain the response to immune checkpoint inhibitors treatment. Current bioinformatics technology enabled us to characterize the immune infiltration pattern and the immune score of tumors. Therefore, we intended to divide STAD samples into hot and cold tumors based on the data in TCGA and to identify signature genes that are associated with the hot and cold tumors for prognostic model establishment.

## 2 Materials and Methods

### 2.1 Data Acquisition

The FPKM RNA-sequencing data and clinical phenotype data of STAD were downloaded from The Cancer Genome Atlas (TCGA) database, including 350 tumor samples and 31 adjacent normal samples. Somatic mutation data of STAD was also acquired from the TCGA database. The preprocessed series matrix of GSE19188 was downloaded from Gene Expression Omnibus (GEO) database, which involves 300 STAD samples and 100 normal samples. Data were annotated based on the annotation files (hg38, V22) provided in the Gencode database, with Ensembl-ID converting to gene symbol. The mean value was considered as the final expression value when multiple Ensembl-ID matched to one gene symbol.

### 2.2 Characterization of Immune Infiltration

The infiltration abundance of 22 immune cells of STAD was estimated by using the CIBERSORT algorithm based on the LM22 gene expression characteristic provided on the CIBERSORT website with parameters set as perm = 100 and QN = F. Samples with *p* < 0.05 were screened for estimating the infiltration landscape of STAD and normal samples. The stromal score, immune score, and tumor purity of STAD samples were calculated by using the ESTIMATE package in R. In addition, the infiltration abundance of six immune cell types was estimated by using the TIMER online tool (https://cistrome.shinyapps.io/timer/).

### 2.3 Consensus Clustering Analysis

Based on the infiltration abundance of 22 immune cells, STAD samples were grouped into different clusters by using the consensuscluster Plust algorithm (version 1.50.0) with parameters set as maxK = 6, pItem = 0.8, clusterAlg = “hc” and distance = “pearson”. The cumulative distribution function (CDF) was used to identify the most reasonable number of clusters.

### 2.4 Hot and Cold Tumors

Based on immune infiltration, immune score, and consensus clustering subtypes, STAD samples were categorized into hot tumors and cold tumors. Kaplan-Meier (KM) survival analysis with log-rank test was performed using the survival package (version3.2–7). Tumor mutation burden (TMB) of hot and cold tumors was analyzed using the Maftools package (version 2.0.16) based on the somatic mutation data.

### 2.5 Differential Expression Analysis and Function Enrichment

The differentially expressed genes (DEGs) between hot tumor and cold tumor were screened based on the *t*-test provided in the Limma package (version 3.10.3) with cut-off values of Benjamini–Hochberg (BH) multiple tests adjusted *p* < 0.05 and |log_2_FC|>0.585. Gene ontology annotation terms and KEGG pathways were enriched for DEGs using the over-representation analysis (ORA) method provided by the gprofiler online tool (https://biit.cs.ut.ee/gprofiler/convert) with a cut-off value of BH adjusted *p* < 0.05.

### 2.6 Co-expression of DEGs With N6-Methyladenosine (m6A) Regulator Genes

Expression of m6A regulator genes (writers: METTL3, METTL14, METTL15, WTAP, VIRMA, RBM15, RBM15B, KIAA1429, ZC3H13; erasers: FTO, ALKBH5; readers: RBMX, YTHDC1, YTHDC2, IGF2BP1, IGF2BP2, IGF2BP3, YTHDF1, YTHDF2, YTHDF3, HNRNPA2B1, HNRNPC) were extracted from TCGA dataset. Then, the Pearson correlation coefficient (PCC) of the expression level of m6A regulator genes and DEGs was calculated based on the cor test in R 3.6.1 (http://77.66.12.57/R-help/cor.test.html) with a cut-off value of the absolute value of correlation coefficient >0.15 and *p* < 0.05.

### 2.7 Construction of Prognostic Risk Model

KM survival analysis was performed for all DEGs with samples dividing into high-expression and low-expression by median value, and genes with log-rank *p* < 0.05 were regarded as prognostic genes. All the STAD samples were randomly divided into the training-set and validation-set with a ratio of 5:5. In training-set, the univariate Cox regression analysis in the survival package was performed for KM prognostic genes and genes with *p* < 0.05 were considered signature genes for model construction. Multivariable Cox regression analysis was used to calculate the prognostic coefficient for signature genes. Then, the prognostic risk model was established according to the formula: Risk score = ∑Coef gene ×Exp gene, of which Coef and Exp refer to the prognostic coefficient and expression value of each signature gene, respectively.

After calculating the risk score, samples were assigned into high- and low-risk groups based on the median risk score. KM survival analysis was performed to detect the survival differences between the two risk groups. GSE19188 was used as an external dataset to validate the prognostic model.

### 2.8 Protein Expression of Signature Genes

In order to investigate the protein expression of signature genes in STAD, the immunohistochemistry images of STAD were acquired from The Human Protein Atlas (http://www.proteinatlas.org) database.

### 2.9 Statistical Analysis

The correlations of immune cells infiltration abundance with overall survival (OS) were analyzed based on univariate Cox regression analysis in the survival package (version3.2–7). The differences in infiltration abundance of each immune cell type between STAD and normal samples were compared using WilcoxTest. The differences in stromal score, immune score, and tumor purity among different immune subtypes were compared by using a *t*-test. The differences in clinical phenotype (including age, gender, TNM stage, pathologic-stage, and tumor grades) between hot and cold tumors were compared using the chi-square test. Univariate and multivariable Cox regression analyses were performed to evaluate the independent predictive value of the prognostic model. Statistical difference was presented with *p* < 0.05.

## 3 Results

### 3.1 Immune Infiltration Landscape in Tumor and Normal Samples

**FIGURE 1:**
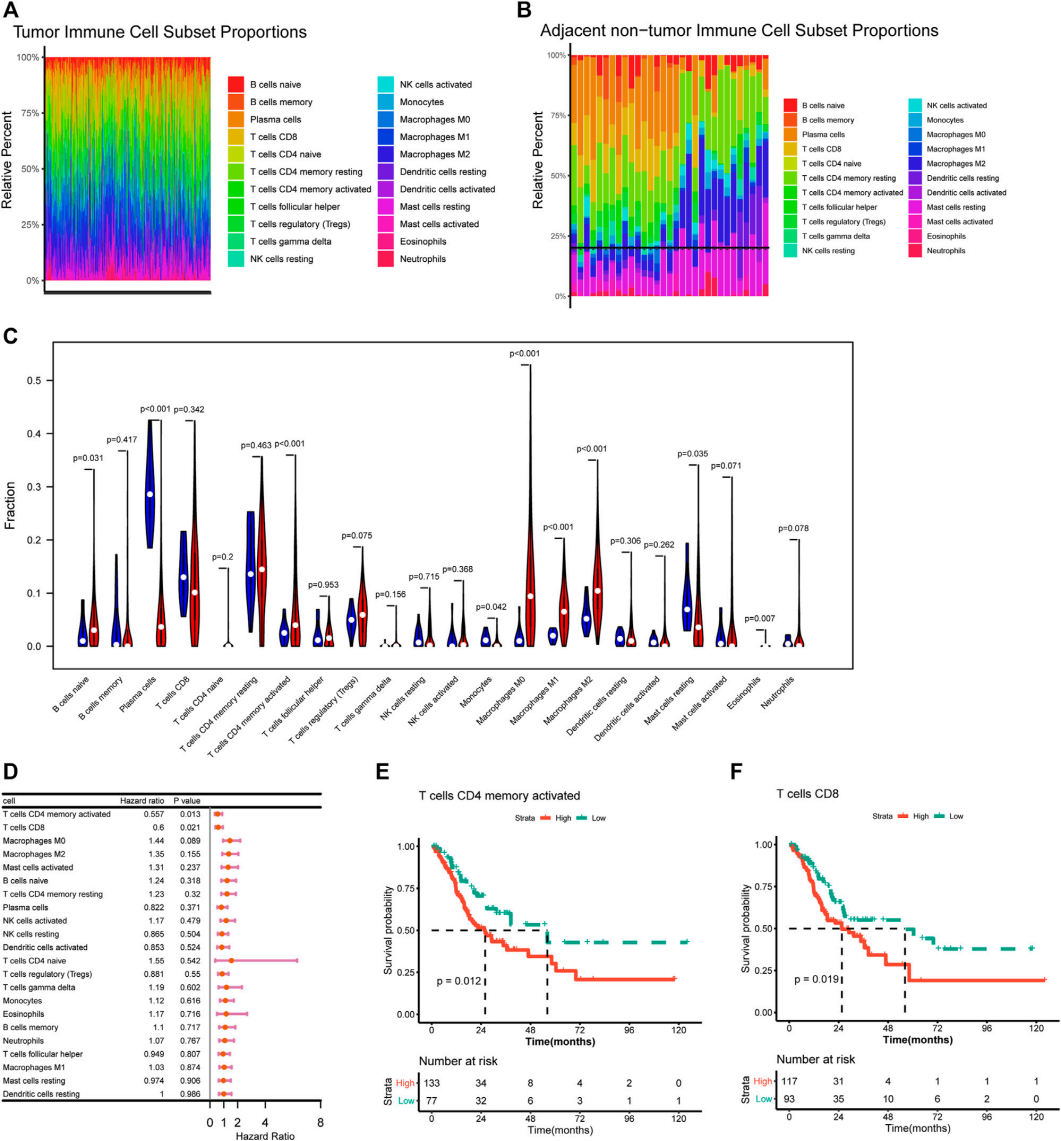
Correlation of immune infiltration and overall survival. **(A–B)** Histogram showed the distribution of 22 immune cells in tumor tissue **(A)** and normal tissue **(B)**. **(C)** Violin plot showed the differences on 22 immune cells infiltration in normal and tumor tissue. **(D)** Forest plot showed the correlation of immune infiltration and overall survival. **(E–F)** Kaplan-Meier survival curves showed the correlation of immune cells infiltration and overall survival.

The proportion of 22 immune infiltration cells in the tumor and normal samples was evaluated. As shown in Figures 1A,B, the infiltration proportion varied among different cell types, and there were also differences in infiltration proportion for the same cell type among samples. The infiltration abundance between tumor and normal samples was also compared. It could be seen that tumor samples showed a significantly higher infiltration abundance of activated memory CD4 T cells, naive B cells, macrophages M0/M1/M2, and eosinophils, while normal samples showed a significantly higher infiltration abundance of plasma cells and resting mast cells (Figure 1C).

### 3.2 Correlations of Immune Infiltration With Overall Survival

The correlations of immune cells infiltration abundance with OS were analyzed based on univariate Cox regression analysis in the survival package, and the results showed that activated memory CD4 T cells (HR = 0.557, *p* = 0.013) and CD8 T cells (HR = 0.6, *p* = 0.021) had significant effect to OS of STAD patients (Figure 1D). Further survival analysis indicated that higher infiltration abundance of activated memory CD4 T cells and CD8 T cells were associated with shorter survival time (Figures 1E,F).

### 3.3 Immune Subtypes of Stomach Adenocarcinomas

**FIGURE 2:**
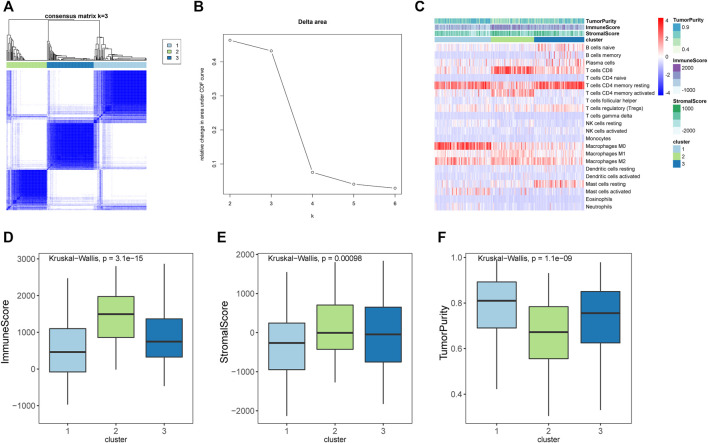
Immune subtypes in stomach adenocarcinomas. **(A)** Heatmap showed the results of consensus clustering analysis, in which samples were divided into three clusters. **(B)** Delta diagram showed the clusters with under area. **(C)** Heatmap showed the immune infiltration pattern of three immune clusters. **(D–F)** The immune score, stromal score, and tumor purity of three clusters.

To explore whether the STAD samples could be grouped into different clusters based on the immune infiltration pattern, a consensus clustering analysis was performed. Results indicated that STAD samples could be grouped into three clusters (Figures 2A,B). The distribution of immunes cells in different clusters were shown in Figure 2C, cluster 2 had significantly higher abundance of CD8 T cells and activated memory CD4 T cells. Cluster 1 had significantly higher abundance of resting memory CD4 T cells and macrophages M0, and cluster 3 had a significantly higher abundance of resting memory CD4 T cells. Further ESTIMATE analysis suggested that cluster 2 had higher immune-score and stromal-score, while showed lower tumor-purity than other clusters (Figures 2D–F). Considering that cluster 2 had higher immune-score and a significantly higher abundance of CD8 T cells and activated memory CD4 T cells, which were important immune cells for targeting cancer in immunotherapy, we assigned cluster 2 as hot tumors. While cluster 1 and 3 had a significantly higher abundance of resting memory CD4 T cells and decreased abundance of CD8 T cells, therefore we assigned cluster 1 and 3 as cold tumors.

### 3.4 Clinical Correlations of Hot and Cold Tumors

**FIGURE 3:**
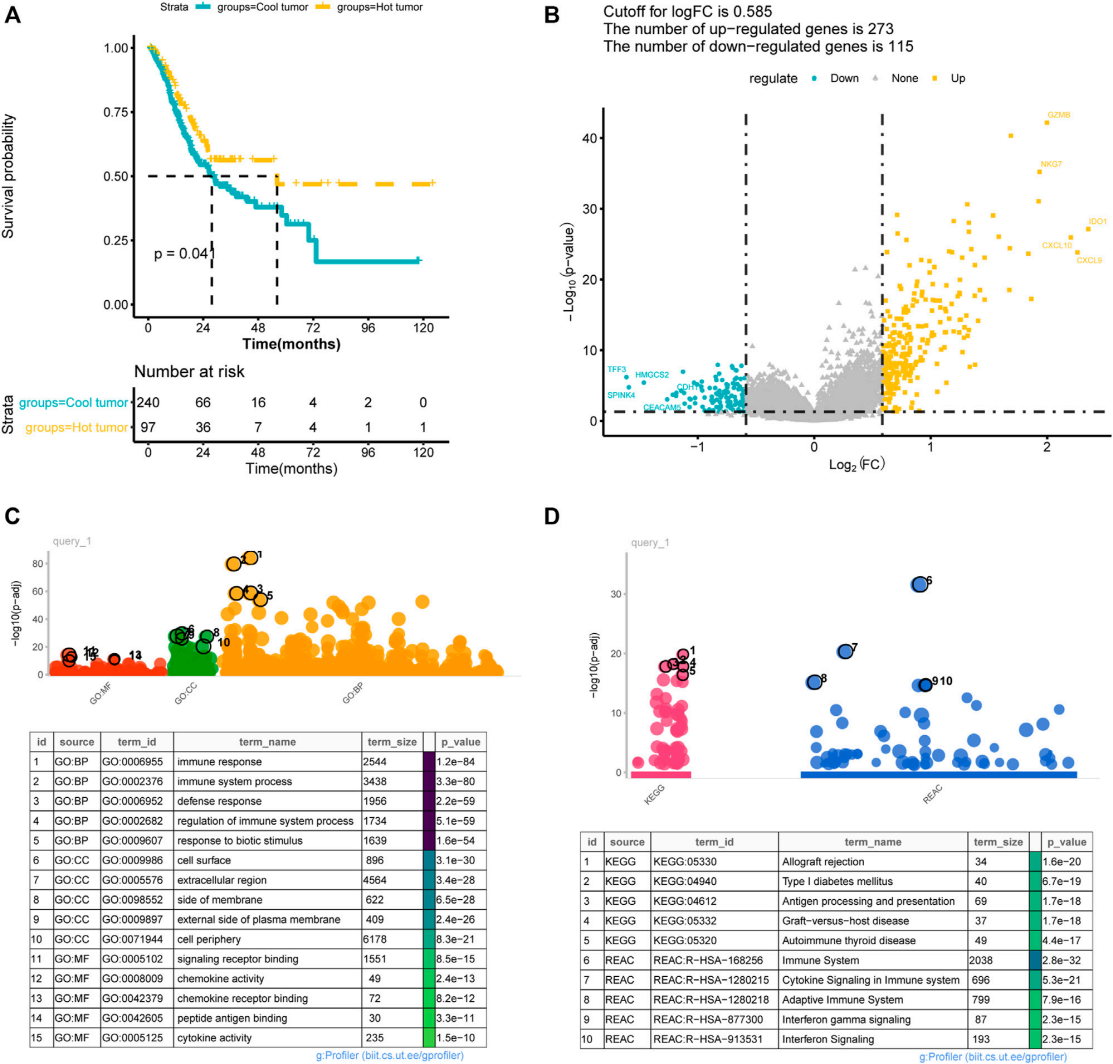
Difference between immune hot and cold tumors. **(A)** Kaplan-Meier curves showed the survival differences between hot and cold tumors; **(B)** volcano plot showed the differentially expressed genes between hot and cold tumors. **(C–D)** The significantly enriched Gene Ontology annotations terms **(C)** and KEGG pathways **(D)**. BP, biological processes; CC, cellular component; MF, molecular function.

The differences in clinical phenotype (including age, gender, TNM stage, pathologic-stage, and tumor grades) between hot and cold tumors were compared using the chi-square test. As shown in Table 1, there were no differences on age, gender, TNM stage, or pathologic-stage between hot and cold tumors, while there had significant differences in tumor grades. The cold tumor contained more proportion of lower grade tumors (grade 1–2, 42.6% vs 22.8%) than the hot tumor, while the hot tumor contained more proportion of higher-grade tumors (grade 3, 72.3% vs 50.6%) than the cold tumor. Survival analysis showed that hot tumors were associated with longer survival (Figure 3A).

**TABLE 1:** The differences on clinical phenotype between hot and cold tumors.

	Cool tumor (N = 249)	Hot tumor (N = 101)	*p*-value
**Gender**			
Female	78 (31.3%)	41 (40.6%)	0.116
Male	162 (65.1%)	56 (55.4%)	
Missing	9 (3.6%)	4 (4.0%)	
**Age (years)**			
<60	73 (29.3%)	32 (31.7%)	0.731
≥60	165 (66.3%)	64 (63.4%)	
Missing	11 (4.4%)	5 (5.0%)	
**Stage**			
Stage I	34 (13.7%)	11 (10.9%)	0.285
Stage II	69 (27.7%)	38 (37.6%)	
Stage III	98 (39.4%)	39 (38.6%)	
Stage IV	27 (10.8%)	7 (6.9%)	
Missing	21 (8.4%)	6 (5.9%)	
**T**			
T1	13 (5.2%)	2 (2.0%)	0.349
T2	56 (22.5%)	18 (17.8%)	
T3	106 (42.6%)	50 (49.5%)	
T4	63 (25.3%)	25 (24.8%)	
Missing	11 (4.4%)	6 (5.9%)	
**N**			
N0	68 (27.3%)	31 (30.7%)	0.24
N1	62 (24.9%)	29 (28.7%)	
N2	55 (22.1%)	13 (12.9%)	
N3	46 (18.5%)	22 (21.8%)	
Missing	18 (7.2%)	6 (5.9%)	
**M**			
M0	215 (86.3%)	88 (87.1%)	0.397
M1	18 (7.2%)	4 (4.0%)	
Missing	16 (6.4%)	9 (8.9%)	
**G**			
G1	9 (3.6%)	0 (0%)	<0.001
G2	97 (39.0%)	23 (22.8%)	
G3	126 (50.6%)	73 (72.3%)	
Missing	17 (6.8%)	5 (5.0%)	

### 3.5 Differential Expression Between Hot and Cold Tumors

**FIGURE 4:**
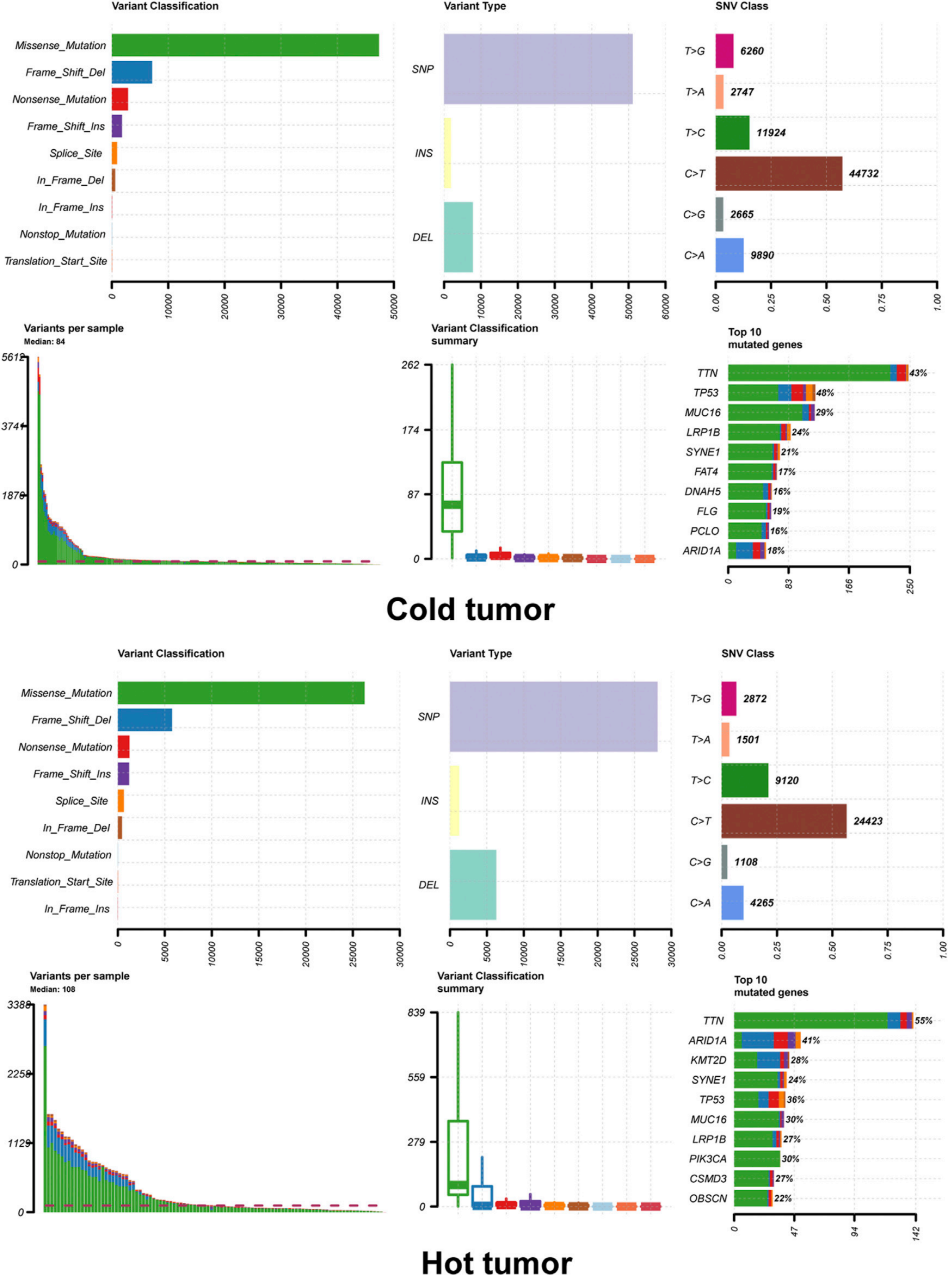
Tumor mutation load of hot and cold tumors. The summary plot of tumor mutation load showed the variant classification, variant type and top 10 mutated genes.

A total of 388 DEGs were screened between hot and cold tumors, of which 273 genes were up-regulated and 115 genes were down-regulated (Figure 3B). Further functional enrichment indicated that these DEGs were significantly involved in immune-related biological processes, such as immune response, immune system process, and regulation of immune system process (Figure 3C). DEGs were also significantly enriched in immune-related pathways, such as antigen processing and presentation; immune system, cytokine signaling in the immune system, adaptive immune system, and interferon signaling (Figure 3D). TMB has been regarded as a prognostic and predictive biomarker for immune checkpoint inhibitors therapy (McNamara et al., 2020). Therefore, we analyzed the TMB patterns, and similar TMB patterns were found between hot and cold tumors (Figure 4). Missense mutation was the most frequent variant classification, single nucleotide polymorphism (SNP) accounted for the most frequent variant types. However, there were significant differences in the top 10 mutated genes and their mutation frequency between hot and cold tumors. The hot tumor had a higher mutation frequency of OBSCN, CSMD3, PIK3CA, and KMT2D, which were not found in the top 10 mutated genes in the cold tumor. The cold tumor had a higher mutation frequency of PCLO, FLG, DNAH5, and FAT4, which were not found in the top 10 mutated genes in the hot tumor. In addition, the mutation frequency for commonly mutated genes showed differences, for example, ARID1A (41% vs 18% in hot and cold tumors).

### 3.6 Identification of Signature Genes for Prognostic Model Establishment

**FIGURE 5:**
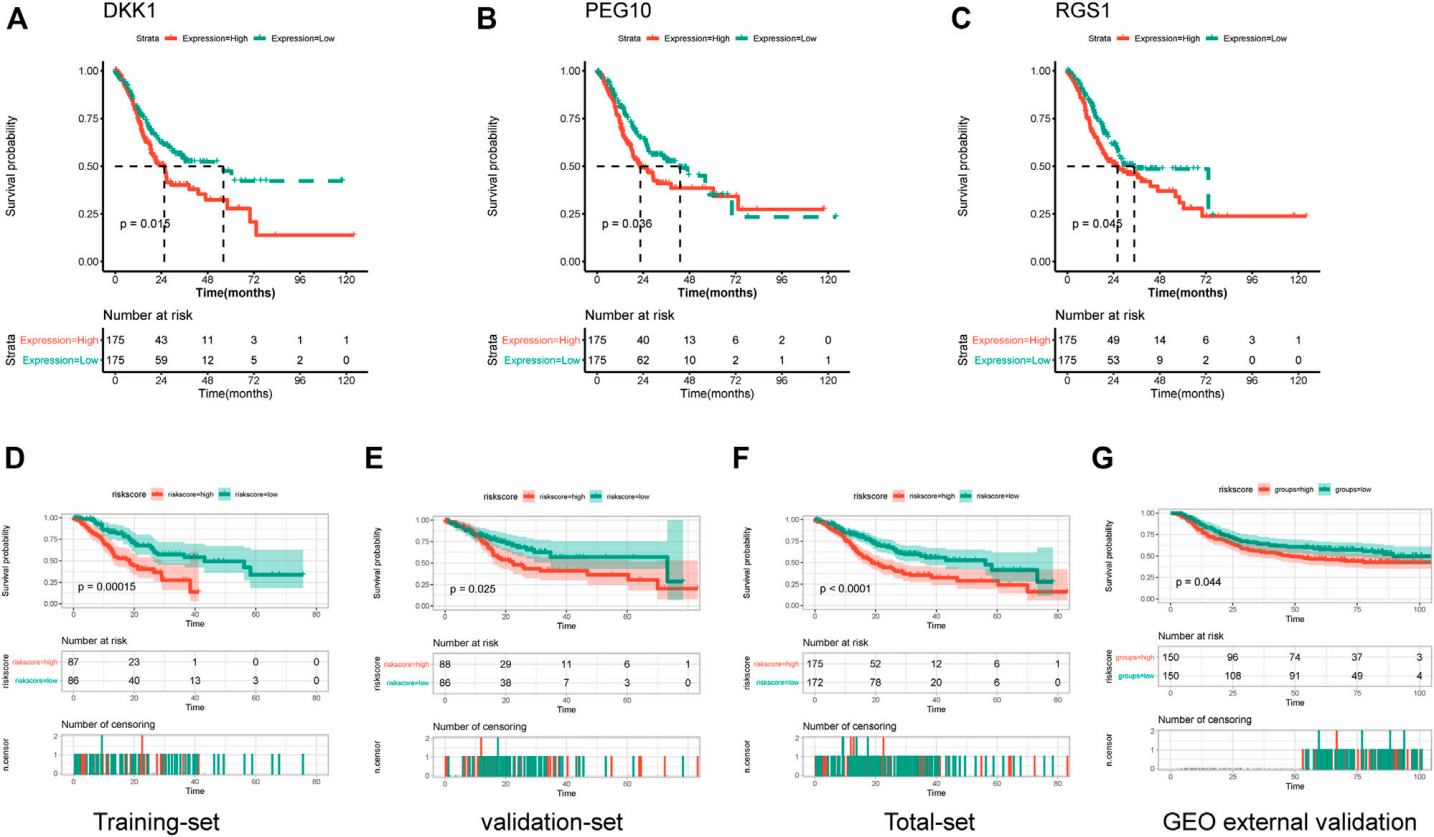
Establishment and validation of prognostic risk model. **(A–C)** Kaplan-Meier curves showed the correlations of genes expression with overall survival. **(D–G)** Kaplan-Meier curves showed the correlations of risk score with overall survival in training-set, validation-set, total-set and GEO external validation set.

In order to investigate the prognostic value of these DEGs, KM survival analysis was performed, and 13 genes were found to be associated with the overall survival of patients (Supplementary Table S1). The associations of these prognostic genes with M6A regulator genes were investigated, and all 13 prognostic genes showed co-expression with m6A regulator genes (Supplementary Figure S1), indicating that these genes might be regulated by m6A methylated modification. Univariate Cox regression analysis was performed for these 13 prognostic genes, and three signature genes (PEG10, DKK1, and RGS1) were identified to have a significant effect on the overall survival of patients (Table 2). These three genes were identified as risk factors for survival with a hazard ratio >1. Consistently, KM survival analysis indicated that high expression of PEG10, DKK1, and RGS1 were associated with poor survival (Figures 5A–C). After calculating the prognostic coefficient for signature genes using multivariable Cox regression analysis, the prognostic model was established with the formula of Risk score = PEG10*0.002 + RGS1*0.018 + DKK1* 0.074. Survival analysis indicated that the risk score could stratify STAD patients, of which patients in the high-risk group were found to have a shorter survival time than that of the low-risk group in both training-set, validation-set, total-set, and GEO external validation dataset (Figures 5D–G). Additionally, the risk score was identified as a prognostic factor independent from other clinical phenotype factors in STAD (Table 3).

**TABLE 2:** Results of univariate Cox regression analysis.

Symbol	Hazard ratio	*p* value
PEG10	1.224(1.091–1.372)	0.001
DKK1	1.161(1.039–1.297)	0.009
RGS1	1.240(1.033–1.488)	0.021
COL10A1	1.124 (0.988–1.278)	0.075
ENTPD8	0.837 (0.688–1.019)	0.076
FUT6	0.873 (0.735–1.039)	0.126
PYCARD	0.812 (0.612–1.079)	0.151
PTPRN2	0.890 (0.749–1.056)	0.182
MICB	0.848 (0.643–1.119)	0.244
BATF2	0.897 (0.745–1.079)	0.248
TK1	0.856 (0.652–1.125)	0.265
MMP12	0.972 (0.871–1.085)	0.617
PSMB10	0.960 (0.746–1.235)	0.75

**TABLE 3:** Univariate and multivariables Cox regression analysis for clinical factors.

Clinical characteristics	Univariables cox		Multivariables cox	
	Hazard ratio	*p* Value	Hazard ratio	*p* Value
pathologic_N	1.328(1.145–1.540)	0	0.107	1.208 (0.960–1.520)
Stage	1.494(1.219–1.830)	0	0.696	1.085 (0.722–1.631)
RiskScore	1.969(1.410–2.751)	0	0.001	1.906(1.310–2.775)
pathologic_T	1.291(1.051–1.586)	0.015	0.381	1.138 (0.852–1.520)
pathologic_M	1.959(1.103–3.481)	0.022	0.523	1.294 (0.586–2.856)
Grade	1.383(1.005–1.904)	0.047	0.394	1.174 (0.812–1.699)
Age	1.446 (0.995–2.102)	0.053		
Groups	0.694 (0.475–1.013)	0.058		
Gender	1.325 (0.930–1.887)	0.119		

### 3.7 Association of Signature Genes Expression With Immune Infiltration Levels

**FIGURE 6:**
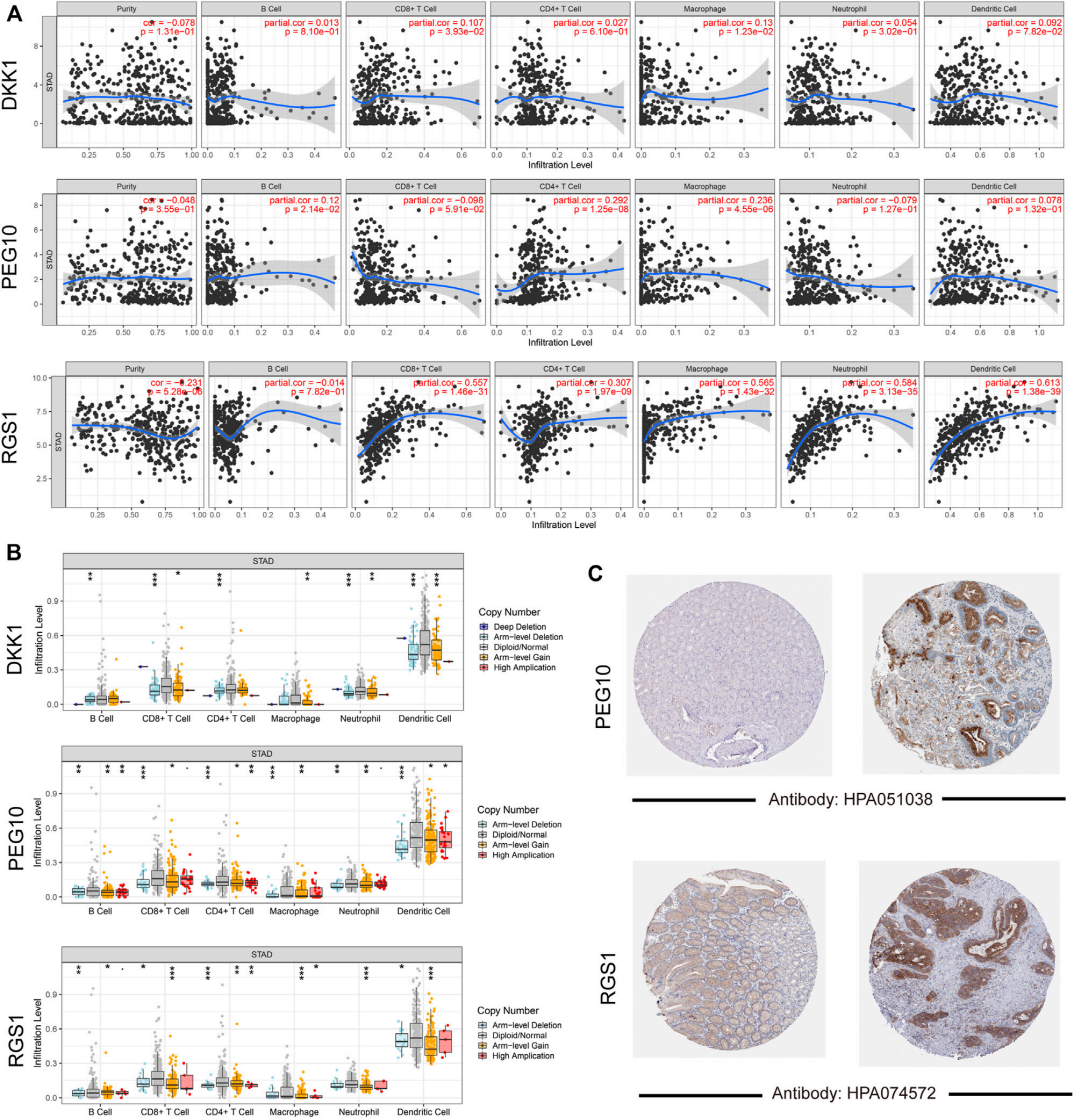
Validation for signature genes. **(A)** Correlations gene expression level with tumor purity and six immune cells infiltration; **(B)** the differences on infiltration levels among different copy number status; **(C)** the immunohistochemistry images showed the protein expression of PEG10 and RGS1.

The associations of signature gene expression with immune infiltration levels in STAD were analyzed using the TIMER online tool. As shown in Figure 6A, expression of RGS1 showed strong positive correlations with the infiltration levels of CD8^+^ T cells, CD4^+^ T cells, macrophage, neutrophil, and dendritic cells (r > 0.3 and *p* < 0.05). PEG10 expression showed weak positive correlations with the infiltration levels of CD4^+^ T cells and macrophages (*p* < 0.05). The infiltration levels of all these six immune cell types showed significant differences among different copy number statuses of three signature genes (Figure 6B). Additionally, to detect the protein expression of signature genes in STAD, the immunohistochemistry images of STAD were acquired from the HPA database. Consistently, protein expression of PEG10 and RGS1 showed significantly high expression in tumor tissue than in the normal tissue (Figure 6C). No immunohistochemistry images for DKK1 were found.

## 4 Discussion

In recent years, the attention to tumor immunotherapy is rapidly increasing, and the theory and practice of immunotherapy for stomach cancer have also achieved good results, bringing hope to patients with advanced cancer, but there are still many obstacles to overcome. For example, some patients showed continued response after a short period of ipilimumab treatment. However, some patients respond well to initial immunotherapy but relapse after a period of time (Bang et al., 2017; Janjigian et al., 2018). Therefore, it is necessary to explore the immune phenotype classification of stomach cancer and screen the dominant population that may benefit from immunotherapy.

In the current study, the immune infiltration landscape of STAD was investigated. Various immune cell types showed significant differences in infiltrating abundance between tumor and normal samples. Tumor samples showed significantly higher infiltration abundance of activated memory CD4 T cells, naive B cells, macrophages, and eosinophils, while normal samples showed significantly higher infiltration abundance of plasma cells and resting mast cells. In addition, activated memory CD4 T cells and CD8 T cells were found to have a significant effect on the OS of STAD patients. This emphasized that the immune infiltrating status had the ability to reflect the prognosis of patients. The purpose of tumor immunotherapy is to promote the activity of cytotoxic T lymphocytes (CTLs) within the tumor and to establish durable and efficient anti-tumor immunity (Maher and Davies, 2004). CD4^+^ T cells can not only kill tumor cells directly in an IFN-γ-dependent manner but also maintain and promote the survival of CD8^+^ T cells through activation of CD8^+^ T cells, generation of memory CTLs response, and so on (Borst et al., 2018). Tumor-infiltrating lymphocytes (TIL) is a heterogeneous lymphocyte group that has an anti-tumor effect on the tumor, and CD8^+^ T cells are the main effector cells (Farhood et al., 2019; Raskov et al., 2021). The loss of TILs function in the tumor microenvironment is the main factor leading to tumor progression and failure of cellular immunotherapy (Zhang et al., 2019; Lin et al., 2020). Therefore, simultaneously activating CD4^+^ T cells and CD8^+^ T cells is an ideal strategy for immunotherapy.

Based on the immune infiltration pattern, the STAD samples were grouped into three clusters in consensus clustering analysis, of which Cluster two was mainly enriched by a higher abundance of CD8 T cells and activated memory CD4 T cells, and had high immune-score calculated by ESTIMATE algorithm. Therefore Cluster 2 was defined as a hot tumor. While cluster 1 and 3 had a significantly higher abundance of resting memory CD4 T cells and decreased abundance of CD8 T cells as well as a lower immune score, therefore we assigned cluster 1 and 3 as a cold tumor. The differences at the transcriptional level between hot and cold tumors were further investigated. Consistently, genes that were differentially expressed between hot and cold tumors were mainly enriched in immune-related biological processes and pathways, such as antigen processing and presentation; immune system, cytokine signaling in the immune system, adaptive immune system, and interferon signaling.

From these DEGs, three signature genes associated with overall survival were identified, including PEG10, DKK1, and RGS1. PEG10 encodes paternally expressed gene 10, and has been demonstrated to be highly expressed in various tumors, functioning as an oncogene involved in apoptosis, proliferation, and metastasis of tumors (Xie et al., 2018). PEG10 expression has been linked to worse prognosis and tumor progression or recurrence in endometrial cancer (Zhang et al., 2021), hepatocellular carcinoma (Bang et al., 2015), oral squamous cell carcinoma (Sharan Singh et al., 2017), showing potential predictive and prognostic ability as a biomarker (Ge et al., 2018). PEG10 knockdown showed an anti-tumor effect in stomach cancer by inhibiting proliferation, migration, and invasion (Wang et al., 2018). DDK1 is a dickkopf-related protein, also known as an inhibitor one of beta-catenin-dependent Wnt signaling. Elevated expression of DKK1 has been reported in different tumors (Betella et al., 2020). Expression of DKK1 in both serum and tissue levels has been reported to be a biomarker in diagnosis and predicting survival and tumor recurrence in stomach cancer (Lee et al., 2012; Liu et al., 2016; Hong et al., 2018). In addition, DDK1 has been linked to T-DM1 resistance in STAD (Li et al., 2018). Moreover, DKN-01, an IgG4 monoclonal antibody targeting DKK1 has been demonstrated to have therapeutic benefits in gastroesophageal malignancies (Wall et al., 2020), indicating the potential of DKK1 in cancer therapy. RGS1, a regulator of G protein signaling 1, expression has been reported to have a significant effect on the survival of stomach cancer, and is associated with the differentiation degree of tumor (Li S. et al., 2021). RGS is linked to various immune-mediated diseases. RGS1 is highly expressed in certain B cells, and deeply affects the directed migration of lymphoid cells (Moratz et al., 2000). Dendritic cells transfected with RGS1 can generate RGS1-specific cytotoxic T cells (Grünebach et al., 2008). Also, RGS1 has been reported to affect the frequency of follicular helper T cells (Caballero-Franco and Kissler, 2016). This was consistent with our findings that expression of RGS1 showed strong positive correlations with the infiltration levels of CD8^+^ T cells, CD4^+^ T cells, macrophage, neutrophil, and dendritic cells. Limitations of our study are as follows: first, our prediction model was constructed and validated with data from the TCGA database and GEO based on the “cold” and “hot” tumors. The use of this model in a real clinical setting remains controversial. More studies are still required to confirm our observation. Besides, the testing result of mRNA expression level is not stable. In the future, we will further verify this prediction model at the protein expression level.

## 5 Conclusion

A three-gene-based immune-related prognostic model was established, which could stratify STAD patients well. Patients with the high-risk score were associated with worse survival, and the risk score had an independent prognostic value. Our model contributed to exploring the immune phenotype classification of STAD and screening the dominant population that may benefit from immunotherapy.

## Data Availability

The original contributions presented in the study are included in the article/Supplementary Material, further inquiries can be directed to the corresponding authors.

## Supplementary Material

Supplementary Figure S1 Expression correlations of prognostic genes and m6A regulator genes. ∗∗p<0.01

Supplementary Table S1 The 13 prognostic related genes identified by Kaplan-Meier survival analysis.

## References

[B1] Bang H. Ha S. Y. Hwang S. H. Park C.-K. (2015). Expression of PEG10 Is Associated with Poor Survival and Tumor Recurrence in Hepatocellular Carcinoma. Cancer Res. Treat. 47, 844–852. 10.4143/crt.2014.124 25687862 PMC4614193

[B2] Bang Y.-J. Cho J. Y. Kim Y. H. Kim J. W. Di Bartolomeo M. Ajani J. A. (2017). Efficacy of Sequential Ipilimumab Monotherapy Versus Best Supportive Care for Unresectable Locally Advanced/Metastatic Gastric or Gastroesophageal Junction Cancer. Clin. Cancer Res. 23, 5671–5678. 10.1158/1078-0432.ccr-17-0025 28655793

[B3] Betella I. Turbitt W. J. Szul T. Wu B. Martinez A. Katre A. (2020). Wnt Signaling Modulator DKK1 as an Immunotherapeutic Target in Ovarian Cancer. Gynecol. Oncol. 157, 765–774. 10.1016/j.ygyno.2020.03.010 32192732

[B4] Borst J. Ahrends T. Bąbała N. Melief C. J. M. Kastenmüller W. (2018). CD4(+) T Cell Help in Cancer Immunology and Immunotherapy. Nat. Rev. Immunol. 18, 635–647. 10.1038/s41577-018-0044-0 30057419

[B5] Bray F. Ferlay J. Soerjomataram I. Siegel R. L. Torre L. A. Jemal A. (2018). Global Cancer Statistics 2018: GLOBOCAN Estimates of Incidence and Mortality Worldwide for 36 Cancers in 185 Countries. CA Cancer J. Clin. 68, 394–424. 10.3322/caac.21492 30207593

[B6] Caballero-Franco C. Kissler S. (2016). The Autoimmunity-Associated Gene RGS1 Affects the Frequency of T Follicular Helper Cells. Genes Immun. 17, 228–238. 10.1038/gene.2016.16 27029527 PMC4892947

[B7] Chen W. Zheng R. Baade P. D. Zhang S. Zeng H. Bray F. (2016). Cancer Statistics in China, 2015. CA Cancer J. Clin. 66, 115–132. 10.3322/caac.21338 26808342

[B8] de Haas S. Delmar P. Bansal A. T. Moisse M. Miles D. W. Leighl N. (2014). Genetic Variability of VEGF Pathway Genes in Six Randomized Phase III Trials Assessing the Addition of Bevacizumab to Standard Therapy. Angiogenesis 17, 909–920. 10.1007/s10456-014-9438-1 25012543

[B9] Farhood B. Najafi M. Mortezaee K. (2019). CD8(+) Cytotoxic T Lymphocytes in Cancer Immunotherapy: A Review. J. Cell Physiol. 234, 8509–8521. 10.1002/jcp.27782 30520029

[B10] Feng R.-M. Zong Y.-N. Cao S.-M. Xu R.-H. (2019). Current Cancer Situation in China: Good or Bad News from the 2018 Global Cancer Statistics? Cancer Commun. 39, 22. 10.1186/s40880-019-0368-6 PMC6487510 31030667

[B11] Gao K. Wu J. (2019). National Trend of Gastric Cancer Mortality in China (2003-2015): A Population-Based Study. Cancer Commun. 39, 24. 10.1186/s40880-019-0372-x PMC6498569 31046840

[B12] Ge H. Yan Y. Wu D. Huang Y. Tian F. (2018). Prognostic Value of PEG10 in Asian Solid Tumors: A Meta-Analysis. Clinica Chim. Acta 483, 197–203. 10.1016/j.cca.2018.04.041 29727698

[B13] Grünebach F. Erndt S. Häntschel M. Heine A. Brossart P. (2008). Generation of Antigen-Specific CTL Responses Using RGS1 mRNA Transfected Dendritic Cells. Cancer Immunol. Immunother. 57, 1483–1491. 10.1007/s00262-008-0486-5 18301890 PMC11031069

[B14] Hong S. A. Yoo S. H. Lee H. H. Sun D. S. Won H. S. Kim O. (2018). Prognostic Value of Dickkopf-1 and ß-Catenin Expression in Advanced Gastric Cancer. BMC Cancer 18, 506. 10.1186/s12885-018-4420-8 29720122 PMC5930854

[B15] Janjigian Y. Y. Bendell J. Calvo E. Kim J. W. Ascierto P. A. Sharma P. (2018). CheckMate-032 Study: Efficacy and Safety of Nivolumab and Nivolumab Plus Ipilimumab in Patients with Metastatic Esophagogastric Cancer. J. Clin. Oncol. 36, 2836–2844. 10.1200/jco.2017.76.6212 30110194 PMC6161834

[B16] Le D. T. Uram J. N. Wang H. Bartlett B. R. Kemberling H. Eyring A. D. (2015). PD-1 Blockade in Tumors with Mismatch-Repair Deficiency. N. Engl. J. Med. 372, 2509–2520. 10.1056/nejmoa1500596 26028255 PMC4481136

[B17] Lee H. S. Lee H. E. Park D. J. Kim H.-H. Kim W. H. Park K. U. (2012). Clinical Significance of Serum and Tissue Dickkopf-1 Levels in Patients with Gastric Cancer. Clinica Chim. Acta 413, 1753–1760. 10.1016/j.cca.2012.07.003 22796372

[B18] Li H. Xu X. Liu Y. Li S. Zhang D. Meng X. (2018). MMP7 Induces T-DM1 Resistance and Leads to the Poor Prognosis of Gastric Adenocarcinoma via a DKK1-Dependent Manner. Anticancer Agents Med. Chem. 18, 2010–2016. 10.2174/1871520619666181203111329 30501604

[B19] Li K. Zhang A. Li X. Zhang H. Zhao L. (2021a). Advances in Clinical Immunotherapy for Gastric Cancer. Biochim. Biophys. Acta Rev. Cancer 1876, 188615. 10.1016/j.bbcan.2021.188615 34403771

[B20] Li S. Yang H. Li S. Zhao Z. Wang D. Fu W. (2021b). High Expression of Regulator of G-Protein Signalling 1 is Associated with the Poor Differentiation and Prognosis of Gastric Cancer. Oncol. Lett. 21, 322. 10.3892/ol.2021.12584 33692854 PMC7933750

[B21] Lin B. Du L. Li H. Zhu X. Cui L. Li X. (2020). Tumor-Infiltrating Lymphocytes: Warriors Fight against Tumors Powerfully. Biomed. Pharmacother. 132, 110873. 10.1016/j.biopha.2020.110873 33068926

[B22] Liu Q.-R. Li Y.-F. Deng Z.-Q. Cao J.-Q. (2016). Prognostic Significance of Dickkopf-1 in Gastric Cancer Survival: A Meta-Analysis. Genet. Test. Mol. Biomarkers 20, 170–175. 10.1089/gtmb.2015.0154 27023747

[B23] Llosa N. J. Cruise M. Tam A. Wicks E. C. Hechenbleikner E. M. Taube J. M. (2015). The Vigorous Immune Microenvironment of Microsatellite Instable Colon Cancer is Balanced by Multiple Counter-Inhibitory Checkpoints. Cancer Discov. 5, 43–51. 10.1158/2159-8290.cd-14-0863 25358689 PMC4293246

[B24] Maher J. Davies E. T. (2004). Targeting Cytotoxic T Lymphocytes for Cancer Immunotherapy. Br. J. Cancer 91, 817–821. 10.1038/sj.bjc.6602022 15266309 PMC2409863

[B25] Maleki Vareki S. (2018). High and Low Mutational Burden Tumors Versus Immunologically Hot and Cold Tumors and Response to Immune Checkpoint Inhibitors. J. Immunother. Cancer 6, 157. 10.1186/s40425-018-0479-7 30587233 PMC6307306

[B26] McNamara M. G. Jacobs T. Lamarca A. Hubner R. A. Valle J. W. Amir E. (2020). Impact of High Tumor Mutational Burden in Solid Tumors and Challenges for Biomarker Application. Cancer Treat. Rev. 89, 102084. 10.1016/j.ctrv.2020.102084 32738738

[B27] Moratz C. Kang V. H. Druey K. M. Shi C.-S. Scheschonka A. Murphy P. M. (2000). Regulator of G Protein Signaling 1 (RGS1) Markedly Impairs Giα Signaling Responses of B Lymphocytes. J. Immunol. 164, 1829–1838. 10.4049/jimmunol.164.4.1829 10657631

[B28] Raskov H. Orhan A. Christensen J. P. Gögenur I. (2021). Cytotoxic CD8(+) T Cells in Cancer and Cancer Immunotherapy. Br. J. Cancer 124, 359–367. 10.1038/s41416-020-01048-4 32929195 PMC7853123

[B29] Rima F. A. Hussain M. Dewan R. K. Haque M. N. Sultana T. Chowdhury F. (2020). Clinicopathologic Features of Gastric and Gastrooesophageal Junction Adenocarcinoma. Mymensingh Med. J. 29, 195–201. 31915358

[B30] Sharan Singh S. Kumar R. Singh Kushwaha V. Bhatt M. L. B. B. Singh A. Mishra A. (2017). Expression of Radioresistant Gene PEG10 in OSCC Patients and its Prognostic Significance. Asian Pac. J. Cancer Prev. 18, 1513–1518. 10.22034/APJCP.2017.18.6.1513 28669160 PMC6373826

[B31] Tabernero J. Hoff P. M. Shen L. Ohtsu A. Shah M. A. Cheng K. (2018). Pertuzumab Plus Trastuzumab and Chemotherapy for HER2-Positive Metastatic Gastric or Gastro-Oesophageal junction Cancer (JACOB): Final Analysis of a Double-Blind, Randomised, Placebo-Controlled Phase 3 Study. Lancet Oncol. 19, 1372–1384. 10.1016/s1470-2045(18)30481-9 30217672

[B32] Taieb J. Moehler M. Boku N. Ajani J. A. Yañez Ruiz E. Ryu M.-H. (2018). Evolution of Checkpoint Inhibitors for the Treatment of Metastatic Gastric Cancers: Current Status and Future Perspectives. Cancer Treat. Rev. 66, 104–113. 10.1016/j.ctrv.2018.04.004 29730461

[B33] van Velzen M. J. M. Derks S. van Grieken N. C. T. Haj Mohammad N. van Laarhoven H. W. M. (2020). MSI as a Predictive Factor for Treatment Outcome of Gastroesophageal Adenocarcinoma. Cancer Treat. Rev. 86, 102024. 10.1016/j.ctrv.2020.102024 32388292

[B34] Vrána D. Matzenauer M. Neoral Č. Aujeský R. Vrba R. Melichar B. (2018). From Tumor Immunology to Immunotherapy in Gastric and Esophageal Cancer. Int. J. Mol. Sci. 20 (1), 13. 10.3390/ijms20010013 PMC6337592 30577521

[B35] Waldman A. D. Fritz J. M. Lenardo M. J. (2020). A Guide to Cancer Immunotherapy: From T Cell Basic Science to Clinical Practice. Nat. Rev. Immunol. 20, 651–668. 10.1038/s41577-020-0306-5 32433532 PMC7238960

[B36] Wall J. A. Klempner S. J. Arend R. C. (2020). The Anti-DKK1 Antibody DKN-01 as an Immunomodulatory Combination Partner for the Treatment of Cancer. Expert Opin. Investig. Drugs 29, 639–644. 10.1080/13543784.2020.1769065 32408777

[B37] Wang J. Chu X. Q. Zhang D. Kong D. F. (2018). Knockdown of Long Non-Coding RNA PEG10 Inhibits Growth, Migration and Invasion of Gastric Carcinoma Cells by Up-Regulating miR-3200. Neoplasma 65, 769–778. 10.4149/neo_2018_171204n794 29940767

[B38] Xie T. Pan S. Zheng H. Luo Z. Tembo K. M. Jamal M. (2018). PEG10 as an Oncogene: Expression Regulatory Mechanisms and Role in Tumor Progression. Cancer Cell Int. 18, 112. 10.1186/s12935-018-0610-3 30123090 PMC6090666

[B39] Zhang D. He W. Wu C. Tan Y. He Y. Xu B. (2019). Scoring System for Tumor-Infiltrating Lymphocytes and its Prognostic Value for Gastric Cancer. Front. Immunol. 10, 71. 10.3389/fimmu.2019.00071 30761139 PMC6361780

[B40] Zhang L. Wan Y. Zhang Z. Jiang Y. Gu Z. Ma X. (2021). IGF2BP1 Overexpression Stabilizes PEG10 mRNA in an m6A-Dependent Manner and Promotes Endometrial Cancer Progression. Theranostics 11, 1100–1114. 10.7150/thno.49345 33391523 PMC7738899

