# Supplementary figures and images for: An Immune-Related Prognostic Signature Predicts Overall Survival in Stomach Adenocarcinomas
Source: Front Genet. 2022 May 23;13:903393. doi: 10.3389/fgene.2022.903393 (PMC9168657; doi:10.3389/fgene.2022.903393)

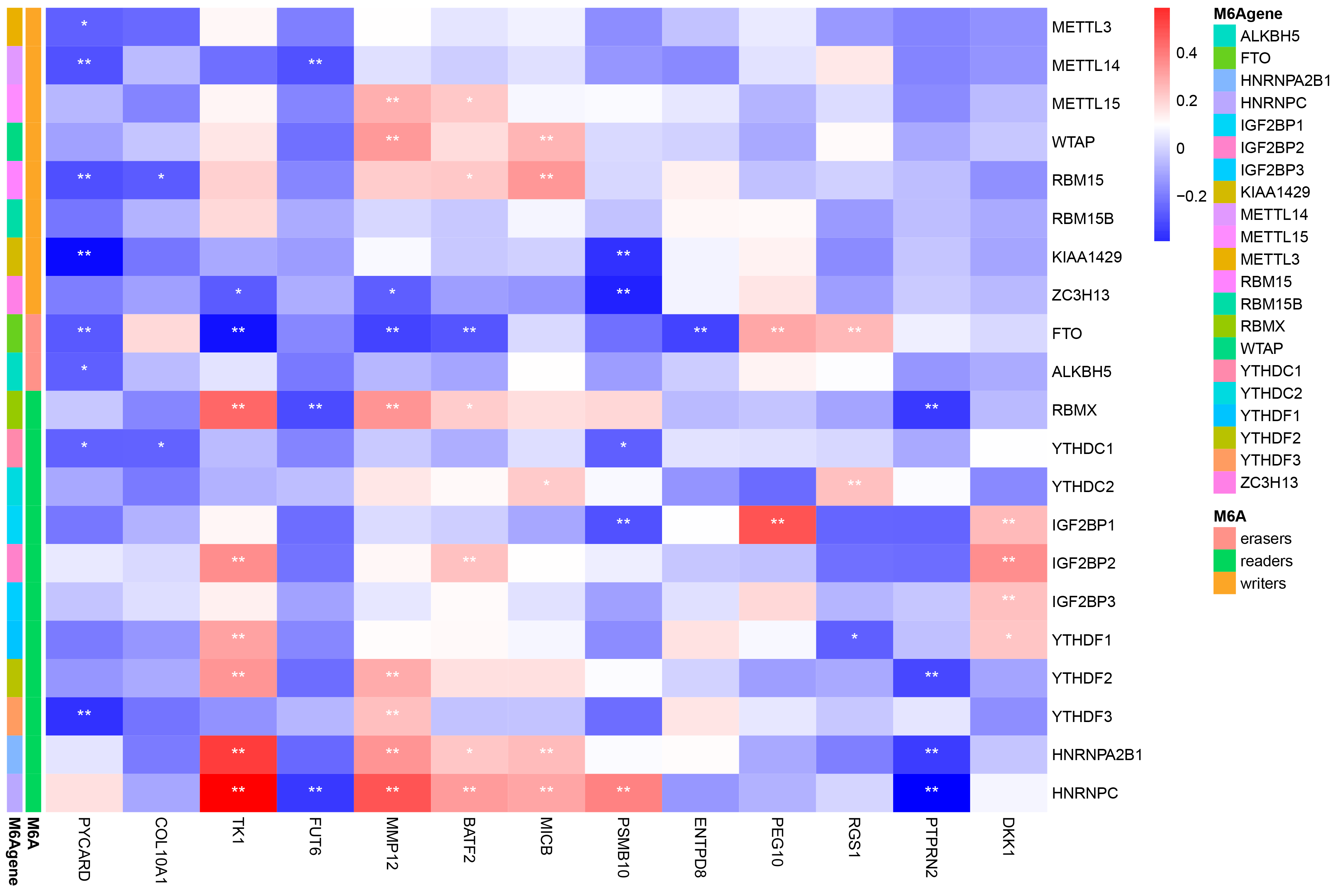

Supplement: Supplementary file 1 [file Image1.TIF]
